# Antifungal and biocontrol potential of *Rhodotorula* carotenoid pigments against mycotoxigenic *Aspergillus* species in cereal-based infant foods

**DOI:** 10.1093/sumbio/qvaf028

**Published:** 2025-10-31

**Authors:** Amina Badmos, Praise Emmanuel, Tolulope Adeleye

**Affiliations:** Department of Microbiology, Federal University of Agriculture, Abeokuta 11011, Nigeria; Department of Environmental Health Sciences, University of Ibadan, Ibadan 20005, Nigeria; Department of Microbiology, Federal University of Agriculture, Abeokuta 11011, Nigeria

## Abstract

This study investigated the occurrence of fungal contaminants in commercially available cereal-based infant foods and explored the potential of pigmented *Rhodotorula* yeast isolates as natural antifungal agents. A total of six fungal isolates were recovered from corn-, rice-, and wheat-based infant foods, representing four genera: *Aspergillus* (*A. niger, A. versicolor, A. terreus*), *Mucor, Rhizopus*, and *Saccharomyces cerevisiae*. Toxigenicity assays revealed that *A. versicolor* and *A. niger* produced mycotoxins, while aflatoxin quantification via HPLC detected total aflatoxin concentrations ranging from 0.56 ± 0.04 µg/g to 9.20 ± 0.02 µg/g across the samples.100 environmental samples yielded 23 pigmented yeasts, from which three distinct *Rhodotorula* strains (YP1, YP2, YP3) were selected based on robust pigment production and growth characteristics. Carotenoid pigments were extracted using acetone and petroleum ether, with storage optimised under dark conditions at 4°C. Protein profiling by SDS-PAGE revealed that all three yeast isolates shared similar banding patterns. Antifungal activity of the yeast-derived pigments against toxigenic *Aspergillus* isolates shows YP3 exhibited the strongest inhibition, producing zones of 21 mm against *A. versicolor* and 19 mm against *A. niger*, more than antifungal agents (fluconazole, ketoconazole, itraconazole, amphotericin B), which showed limited activity (1–5 mm inhibition zones).

**Sustainability Statement**

This study supports SDG 3 (Good Health and Well-being) by addressing food safety risks associated with fungal contamination and mycotoxin exposure in cereal-based infant foods. The discovery of pigmented *Rhodotorula* yeast as a natural antifungal agent offers a safer alternative to synthetic preservatives, contributing to the reduction of foodborne diseases and long-term health risks. By promoting natural bio-preservatives, this research also aligns with sustainable food production practices.

Additional relevant SDGs: SDG 2 (Zero Hunger), SDG 12 (Responsible Consumption and Production).

## Introduction

Food quality and safety have been of paramount concern to humanity throughout history. Contamination of crops during pre- and post-harvest by toxigenic fungi and the consequent production of mycotoxins present major challenges to food safety and human and animal health (Bhat et al. 2022). Mycotoxins are secondary toxic metabolites synthesised by fungi, enabling them to secure an ecological advantage and adapt to extreme environmental conditions (Ismail et al. 2021). Among all mycotoxins, aflatoxins are considered the most important due to their severe health risks to animals and humans. These toxic metabolites are primarily produced by species such as *Aspergillus flavus, Aspergillus parasiticus*, and *Aspergillus nomius*, naturally occurring fungi that contaminate various substrates (Oladipo et al. 2021).

Over 14 chemical forms of aflatoxins have been identified, with aflatoxins B1, B2, G1, and G2 being the most significant due to their potent toxicity. Their nomenclature is based on their fluorescence under ultraviolet light: blue (B) and green (G) (Wang et al. 2021). Health impacts of aflatoxin ingestion can be acute or chronic, depending on the exposure level, resulting in liver damage, encephalopathy, child stunting, immune suppression, neurological impairment, and infertility in men (Nleya et al. 2021). Particularly concerning is the detection of aflatoxin residues in the brains of children who consumed contaminated maize-based gruels, correlating with incidences of kwashiorkor and high child mortality rates in regions such as Nigeria (Afolabi et al. 2021). Recognising its carcinogenic potential, aflatoxin B1 has been classified as a Group 1 carcinogen by the International Agency for Research on Cancer (IARC) (IARC 2022). Other documented health effects include genotoxicity, estrogenic effects, nephrotoxicity, and teratogenicity

A wide range of food products is susceptible to aflatoxin contamination, including cereals (e.g. maize, rice, wheat, millet, oats), spices, legumes, nuts, oilseeds, fruits, vegetables, dried fruits, cocoa, coffee beans, herbs, and processed foods such as bread, meat, wine, beer, and peanut butter (Adetunji et al. 2020, Tesfaye et al. 2021). Given the central role of cereal-based foods in infant diets, and considering infants' higher consumption-to-body-weight ratio and restricted diets, the presence of aflatoxins in infant cereals poses a significant health risk (Gong et al. 2020). Cereals such as rice, maize, and wheat are staple ingredients in infant foods, thus, contamination of raw materials can easily transfer into the final product, exacerbating health risks (Omotayo et al. 2020).

Given the pressing need for safer food alternatives, biocontrol methods have gained attention. Yeasts are unicellular, eukaryotic fungi known for their adaptability and production of antimicrobial compounds that inhibit the growth of harmful moulds and bacteria (Xu et al. 2020). Species within the genus *Rhodotorula* have shown great promise as antagonistic yeasts, capable of biocontrol against mycotoxigenic fungi (Zhang et al. 2020). *Rhodotorula glutinis*, for instance, has been successfully used to control post-harvest diseases such as strawberry grey mould (*Botrytis cinerea*) (Shi et al. 2021). These pigmented yeasts, widely distributed in soil, air, and even as part of human microbiota, belong to the family Sporidiobolaceae within the Basidiomycota phylum (Liu et al. 2020).

A distinctive feature of *Rhodotorula* species is their ability to produce carotenoids, natural pigments responsible for yellow, orange, red, and purple colouration. Carotenoids have essential biological functions, including serving as precursors to vitamin A, scavenging reactive oxygen species, enhancing immune responses, and exerting anti-inflammatory properties (Saini et al. 2020). These pigments also show strong antioxidant activity, contributing to reduced risks of chronic diseases such as cancer, cardiovascular diseases, and neurodegenerative disorders (Fu et al. 2021). Carotenoids from microbial sources offer significant advantages over plant-derived carotenoids, such as higher yield, less seasonal variation, and easier scalability for industrial applications (Dutta et al. 2021).

Microbial production of carotenoids is gaining traction as a sustainable alternative to synthetic colourants, with *Rhodotorula* species capable of producing β-carotene, torulene, and torularhodin, carotenoids with important applications in the food, pharmaceutical, and cosmetics industries (Martins et al. 2021). These natural pigments are increasingly used not only as colourants but also for their health-promoting properties, such as improving sperm health, supporting liver and prostate health, boosting cognitive function, and enhancing skin health (Ramos et al. 2020). In particular, microbial carotenoids offer an eco-friendly solution to meet the rising global demand for natural additives and bioactive compounds (Molino et al. 2020).

Thus, this study explores the potential of *Rhodotorula* species to serve both as a biocontrol agent against aflatoxigenic fungi in infant cereal-based foods and as a source of bioactive carotenoids. By leveraging the antimicrobial and health-enhancing properties of *Rhodotorula*-derived carotenoids, this research aims to provide an innovative and sustainable approach to mitigating fungal contamination and reducing aflatoxin levels in commercial infant cereals, contributing significantly to improving food safety and public health.

## Materials and methods

### Sample collection

Commercially available infant cereal-based foods (including maize, rice, and wheat-based formulations) were randomly purchased from major retail outlets in Abeokuta, Ogun state, Nigeria. Samples were transported to the laboratory under aseptic conditions and stored at 4°C until further analysis.

### Isolation of fungi

Each cereal sample (10 g) was homogenised in 90 ml of sterile peptone water (0.1% w/v) and serially diluted. Aliquots (0.1 ml) from appropriate dilutions were spread on Potato Dextrose Agar (PDA) plates supplemented with 0.01% chloramphenicol to inhibit bacterial growth. Plates were incubated at 28°C for 5–7 days. Fungal colonies exhibiting morphological characteristics typical of *Aspergillus* spp. were sub-cultured for purification and further identification as described by Samson et al. (2004).

### Toxigenicity Test of fungal isolates

The toxigenicity of *Aspergillus* isolates was assessed following the method described by Atanda et al. (2011). Isolates were cultured on Neutral Red Desiccated Coconut Agar Medium and incubated in a dark cupboard at room temperature for three days. Plates were then exposed to UV light at 360 nm for 3 hrs. Isolates that exhibited fluorescence under UV light were considered aflatoxigenic.

### Molecular identification of isolated *Aspergillus* species

Molecular characterisation of *Aspergillus* isolates was carried out at the International Institute of Tropical Agriculture (IITA), Ibadan, Nigeria. Pure cultures were initially sub-cultured on Potato Dextrose Agar (PDA) and then transferred into Potato Dextrose Broth (PDB), followed by incubation at 28 °C for 48 hours to promote mycelial growth. Mycelial mats were harvested by centrifugation, and genomic DNA was extracted using a modified cetyltrimethylammonium bromide (CTAB) method. 600 µL of 2 × CTAB buffer and 100 µL of 20% SDS were added to the fungal pellet and incubated at 65 °C for 20 minutes. An equal volume of chloroform was added, gently mixed, and centrifuged to separate phases. The upper aqueous layer was transferred to a new tube, and DNA was precipitated using ice-cold isopropanol. The DNA pellet was washed with 70% ethanol, air-dried, and resuspended in 200 µL of sterile distilled water. DNA purity and concentration were assessed using a NanoDrop spectrophotometer and confirmed via 1% agarose gel electrophoresis.

The internal transcribed spacer (ITS) region of the rDNA, which is commonly used for *Aspergillus* species identification, was amplified using the universal primers ITS1 (5′-TCCGTAGGTGAACCTGCGG-3′) and ITS4 (5′-TCCTCCGCTTATTGATATGC-3′). PCR was performed in a final volume of 20 µL containing 60–80 ng of genomic DNA, 0.1 µM of each primer, 2 mM MgCl₂, 125 µM of each dNTP, and 1 U of Taq DNA polymerase. The PCR cycling conditions were as follows: initial denaturation at 94 °C for 3 minutes; 35 cycles of denaturation at 94 °C for 40 seconds, annealing at 52 °C for 50 seconds, and extension at 72 °C for 60 seconds; with a final extension at 72 °C for 5 minutes. Amplified PCR products were resolved on 1.4% agarose gels stained with ethidium bromide (5 mg/mL) and visualised under UV illumination. The amplicons were purified using sodium acetate precipitation and subjected to Sanger sequencing using an ABI 3100 genetic analyser. The resulting nucleotide sequences were compared to reference sequences in the NCBI GenBank database using the BLAST algorithm to determine species identity based on the highest sequence similarity.

### Quantitative analysis for aflatoxins

Aflatoxins were extracted from 25 g of cereal sample using 250 mL of acetone–water (85:15, v/v) and stirred for 45 minutes, following the method described by Shephard (2017) et al. (2017). After filtration, 5 mL of the extract was diluted with 45 mL of distilled water and purified using an Easi-Extract® Aflatoxin immunoaffinity column (R-Biopharm, Glasgow, UK). The column was washed with distilled water, and aflatoxins were eluted with 2.5 mL of methanol. The eluate was evaporated to dryness under a stream of nitrogen, reconstituted in acetonitrile–water (25:75, v/v), filtered through a 0.45 µm Millipore filter, and analysed by High-Performance Liquid Chromatography (Jasco Corporation, Tokyo, Japan) on a Superspher RP-18 column. Detection of aflatoxins was carried out post-column using photochemical derivatisation (UVE, LCTech GmbH) with fluorescence detection at an excitation wavelength of 365 nm and emission at 440 nm.

### Isolation of *Rhodotorula* species

*Rhodotorula* spp. were isolated from different environmental samples, including air, water, soil, fruits, and fermented foods, using Sabouraud Dextrose Agar (SDA).

- Air Samples: Plates containing SDA were exposed to the atmosphere for 3–12 hours, then incubated at 28°C for 24 h.
- Water Samples: Water samples from various locations in Abeokuta were plated using the pour plate method on SDA and incubated at 28°C for 24 h.
- Soil Samples: One gram of soil was serially diluted to 10^⁻6^, and plated on SDA, then incubated at 28°C for 24 h.
- Fruit Samples: Fruits were either rolled over SDA plates or juices were spilt onto the surface before incubation at 28°C for 24 h.
- Fermented Foods: Samples were diluted in sterile water, and appropriate dilutions were plated on SDA, incubated at 28°C for 48 hours.

Distinct pigmented colonies (pink to red) were selected and purified.

### Microscopic identification of *Rhodotorula* species

Yeast isolates were stained using Gram’s staining and lactophenol blue and observed under a light microscope at 40x magnification. Cell morphology, including size and budding patterns, was documented.

### Screening for retention of pigmentation

Visual assessment of pigmentation stability was performed during repeated subculturing and storage of Rhodotorula isolates on slants at 4°C, as described by Pandey et al. (2018).

### Carotenoid extraction

Pigment extraction was done according to the method of Pfander (1992). *Rhodotorula* isolates were grown in glucose-yeast-peptone broth for 10 days. Cells were harvested by centrifugation at 4000 rpm for 5 minutes. The pellet was hydrolysed by adding 1 ml of 5 M HCl and heating at 60°C for 45 minutes. After acid hydrolysis, the pellet was washed thrice with sterile distilled water and resuspended in acetone. The mixture was refrigerated overnight at 4°C. Carotenoids were separated using a sterilised separating funnel, and the pigment layer was collected into sterile centrifuge tubes for further use.

### Protein profile *Rhodotorula* sp. by SDS-PAGE

The selected *Rhodotorula* isolates with the most enhanced carotenoid production were subjected to Sodium Dodecyl Sulphate Polyacrylamide Gel Electrophoresis (SDS-PAGE) to analyze their protein profile. The isolate was cultured in yeast peptone dextrose broth at 28°C for 48 hours. The cells were harvested by centrifugation at 4000 rpm for 10 minutes and washed twice with phosphate-buffered saline (PBS). The cell pellet was resuspended in lysis buffer (containing Tris-HCl, SDS, and β-mercaptoethanol) and incubated at 95°C for 5 minutes to lyse the cells and denature proteins. Protein extracts were loaded onto a 12% polyacrylamide gel. Electrophoresis was carried out at 100 V until the dye front reached the bottom of the gel. After electrophoresis, the gel was stained with Coomassie Brilliant Blue R-250 for 1 hour and destained using a destaining solution (methanol: acetic acid: water in the ratio 4:1:5) until clear bands were visible. The molecular weight of the separated proteins was estimated by comparing them to a standard protein molecular weight marker. The banding pattern was analysed to assess protein diversity and to distinguish the selected isolate from others.

### Evaluation of antifungal activity of carotenoid pigments

Carotenoid pigments dissolved in acetone were tested for antifungal activity using the well diffusion method on Malt Extract Agar plates. Toxigenic mould broth cultures (0.1 mL) were seeded into molten, cooled malt extract agar before solidification. After solidification, wells were punched into the agar, and 1 drop of carotenoid pigment solution was added to each well. Control plates were maintained without pigments. Plates were incubated at room temperature for 72 h, and antifungal activity was determined by measuring the diameter of zones of inhibition around the wells.

### Statistical analysis

All experiments were conducted in triplicate. Data were presented as mean ± standard deviation (SD). Statistical analyses were performed using one-way ANOVA followed by Tukey’s multiple comparison test (*P* < 0.05) using SPSS version 26.0 (IBM Corp., USA).

## Results

### Fungal isolates from cereal-based infant foods

Table 1 presents the results of fungal isolation from cereal-based infant food samples. A total of six fungal isolates were identified, belonging to four genera: *Aspergillus* (including *A. niger, A. versicolor*, and *A. terreus*), *Mucor* spp., *Rhizopus* spp., and *Saccharomyces cerevisiae*.

**Table 1.** Fungal counts and probable organisms from the cereal-based infant foods

Sample code	Fungal count (cfu/g)	Probable organism
CBC	21.0 × 10^5^	*Aspergillus versicolor*
	11.0 × 10^5^	*Mucor spp*.
RBC	32.0 × 10^5^	*Aspergillus niger*
	6.0 × 10 ^5^	*Saccaromyces cerivisae*
	15.0 × 10^5^	*Rhizopus spp*.
WBC	6.0 × 10^5^	*Aspergillus terreus*

CBC: Corn Based Cereal RBC: Rice Based Cereal WBC: Wheat Based Cereal

### Macroscopic and microscopic characteristics of fungal isolates

Table 2 details the macroscopic and microscopic characteristics of fungal isolates obtained from cereal-based infant food samples. A total of five moulds and one yeast were identified. Each isolate exhibited distinct macroscopic features, including colony texture, pigmentation, and growth pattern. Microscopic examination revealed characteristic morphological structures, notably the conidiophores bearing asexual spores (conidia), as well as the structural attributes of the hyphae.

**Table 2.** Macroscopic and microscopic characteristics of fungi isolated from the cereal-based infant foods

Isolate	Microscopy	Macroscopy	Organism
1	Non-septate hyphae, smooth conidiospore, radiate head,biserate sterigmata	Dark green colonies with white edges	*Aspergillus versicolor*
2	Non-septate hyphae without rhizoids	Cotton like colonies	*Mucor spp*
3	Non- septate hyphae, Large/globose condiospores	Black filamentous colonies	*Aspergillus niger*
4	Gram positive budding yeast cell	Creamy white colonies	*Saccaromycescerivisae*
5	Non-septate hyphae with rhizoids	Cotton-like colonies	*Rhizopus spp*.
6	Branched septate hyphae, biserate phalides, smooth conidia	Beige, entire, umbonate and wooly	*Aspergillus terreus*

### Toxigenicity assessment of fungal isolates

Table 3 presents the results of the toxigenicity analysis conducted using Neutral Red Desiccated Coconut Agar. The fungal isolates were incubated for three days at room temperature. Fluorescence under ultraviolet light indicated the production of mycotoxins. Two isolates *Aspergillus versicolor* and *Aspergillus niger* exhibited fluorescence, suggesting their toxigenic potential.

**Table 3.** Toxigenicity test of fungal isolates

Isolate	Organism	Toxigenicity
1	*Aspergillus versicolor*	Toxigenic
3	*Aspergillus niger*	Toxigenic
6	*Aspergillus terreus*	Non-toxigenic

### Molecular identification of aspergillus isolates

Molecular characterization based on ITS region sequencing confirmed the morphological identification of the *Aspergillus* isolates. BLASTn analysis of the ITS sequences revealed high similarity (≥99%) with reference sequences in the NCBI GenBank database, validating the identities of the isolates as *A. flavus, A. niger*, and *A. oryzae* (Table 4).

**Table 4.** Molecular identification of aspergillus isolates

Isolate code	Identified organism (morphology)	Closest GenBank match (BLASTn)	% Identity	E-value	GenBank accession no.	Final identification	Toxigenicity
1	*Aspergillus versicolor*	*Aspergillus flavus* NR_111041.1	100	0	NR_111041.1	*Aspergillus flavus*	Toxigenic
3	*Aspergillus niger*	*Aspergillus niger* MN788109.1	100	0	MN788109.1	*Aspergillus niger*	Toxigenic
6	*Aspergillus terreus*	*Aspergillus oryzae* KC466529.1	99.66	0	KC466529.1	*Aspergillus oryzae*	Non-toxigenic

### Aflatoxin quantification and extraction from cereal-based infant foods

Table 5 presents the concentrations of aflatoxins B₁, B₂, G₁, and G₂ detected in commercially available cereal-based infant food products, as determined using High-Performance Liquid Chromatography (HPLC). The total aflatoxin content across the samples ranged from 0.56 ± 0.04 µg/kg to 9.20 ± 0.02 µg/kg.

**Table 5.** Aflatoxins quantification cereal food sample (μg/g)

Samples	AFB1	AFB2	AFG1	AFG2	Total Aflatoxin	Tolerance limit
**CBC**	4.88 ± 0.00^c^	4.32 ± 0.02^a^	**-**	**-**	9.**20** ± 0.02^c^	0.004
**RBC**	3.86 ± 0.01^b^	**-**	**-**	**-**	3.86 ± 0.01^b^	0.004
**WBC**	0.56 ± 0.04^a^	**-**	**-**	**-**	0.56 ± 0.04^a^	0.004

abcdMeans (±Standard deviation) in the same column having similar superscripts are not significantly different at *P* < 0.05,

Legal tolerance limits4 µg/kg (= 0.004 µg/g) for total aflatoxins and 0.5 µg/kg (= 0.0005 µg/g) for AFB₁, based on Codex Alimentarius (CAC/RCP 45–1993), European Commission Regulation (EC) No 1881/2006, and NAFDAC guidelines.

### Isolation and screening of yeast isolates

A hundred (100) samples were processed as shown in Table 6, which included soil, air, fruits, fermented foods and water. Out of these, 23 pigmented yeasts were isolated. Of the 23 pigmented yeasts isolated, 3 strains of yeasts were selected as they showed distinct pigment production, faster growth as compared to other isolates and the ability to grow on Sabouraud’s agar

**Table 6.** Isolation of *Rhodotorula* species

S/N	Samples processed	No. of samples	Pigmented yeast obtained
1	Air	15	3
2	Water • Rain • Tap	10 10	2 3
3	Soil	15	5
4	Fruits • Orange • Pineapple • Lutea	10 10 10	2 2 1
5	Fermented foods • Cheese • Yoghurt	10 10	3 2
Total		100	23

**Table 7.** Inhibition zone diameters (mm) showing the antifungal activity of selected *Rhodotorula* isolates and standard antifungal drugs against *Aspergillus versicolor*

Treatment	Mean (mm)	SD (mm)	Variance (mm²)
YP1	16.17	6.52	42.48
YP2	10	2.65	7
YP3	21	1	1
Positive control (Keto)	3.33	1.53	2.33
Negative control	0	0	0

**Table 8.** Inhibition zone diameters (mm) showing the antifungal activity of selected *Rhodotorula* isolates and standard antifungal drugs against *Aspergillus niger*.

Treatment	Mean (mm)	SD (mm)	Variance (mm²)
YP1	18	2.65	7
YP2	19.33	1.53	2.33
YP3	19	2	4
Ketoconazole	3.33	1.53	2.33
Fluconazole	5	1	1
Negative Ctrl	0	0	0

### Carotenoid extraction

**Figure 1:**
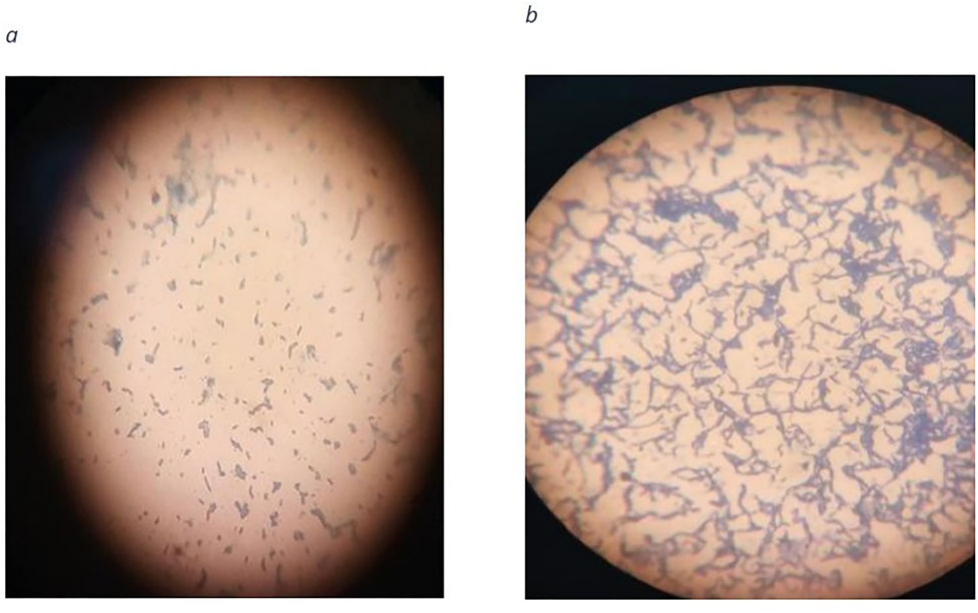
. **aand b show the microscopic view of the *Rhodotorula* Isolates** Microscopic images showing the cellular morphology of *Rhodotorula* species. The yeast cells appear round to oval with characteristic pinkish pigmentation, indicative of the genus *Rhodotorula*.

Pigment extraction from the yeast isolates as shown in (Fig 1a and b )was performed following the method described by Pfander (1992). Pigments were extracted using acetone, partitioned into petroleum ether using a separating funnel. Based on the spectrophotometric analysis reported by Latha and Jeevaratnam (2005), it was established that approximately 35% of the pigments remained stable in petroleum ether after 90 days when stored in the dark at 4°C. Accordingly, all pigment extracts were stored under dark conditions at 4°C in refrigeration until further use. The extracted pigments were then employed for characterisation studies and antifungal assays.

### Protein profile of selected isolates

**Figure 2.**
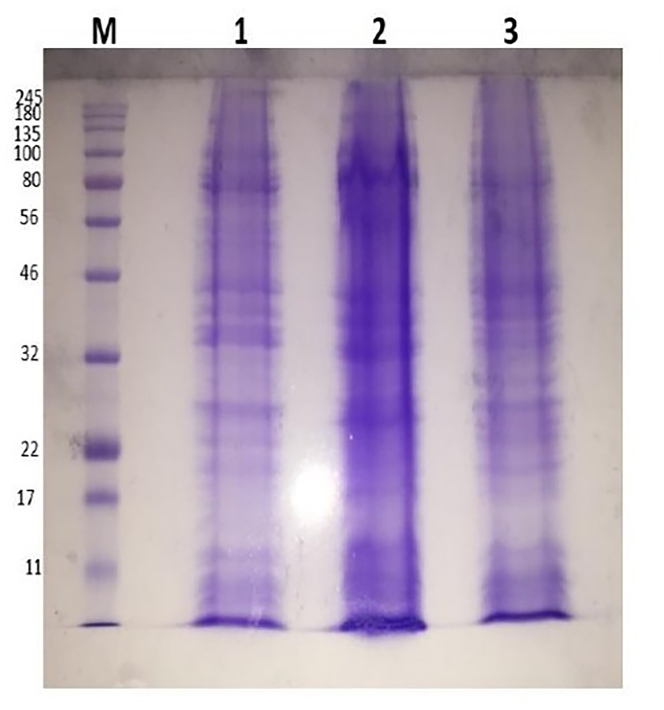
SDS-PAGE gel showing protein profiles of three *Rhodotorula* isolates. All lanes exhibit similar banding patterns, but lane 3 displays a distinct 17 kDa band absent in lanes 1 and 2, indicating possible species variation.

The selected *Rhodotorula* isolates were subjected to SDS-PAGE as described by Laemmli (1970), revealed similar banding patterns with slight variations, suggesting that the isolates belong to the same genus but represent different species. Notably, a distinct protein band at approximately 17 kDa was observed in lane 3, which was absent in lanes 1 and 2, as shown in Fig. 2

### Antifungal assay

The antifungal activity assay demonstrated that the *Rhodotorula* isolates pigments (YP1, YP2, YP3) exhibited notably larger inhibition zones against *Aspergillus versicolor* and *Aspergillus niger* compared to the standard antifungal drugs tested (fluconazole, ketoconazole, itraconazole, amphotericin B) as shown in Table 7 and 8. Specifically, YP3 showed the highest activity, producing inhibition zones of 21 mm (Table 7) and 19 mm ( Table 8) against *A. versicolor* and *A. niger*, respectively. In contrast, the antifungal drugs displayed minimal activity, with inhibition zones generally ranging from 1–5 mm.

## Discussion

The study investigates the occurrence of fungal contamination in cereal-based infant foods and evaluates the potential of *Rhodotorula* yeast pigments as biocontrol agents against toxigenic *Aspergillus* species. The fungal species isolated from infant cereal-based foods, *Aspergillus niger, A. terreus, A. versicolor, Mucor* spp., *Rhizopus* spp., and *Saccharomyces cerevisiae*, are consistent with previous reports on fungal contaminants in food products (Adebayo-Tayo and Oscar 2012). *Aspergillus* species, particularly *A. niger* and *A. versicolor*, are known to produce mycotoxins such as aflatoxins, which pose significant health risks, particularly to infants (Iqbal et al. 2009, Atanda et al. 2011).

The presence of *Aspergillus* species in cereal-based infant foods is likely due to contamination at various stages of food production, including raw material handling, storage, and processing (Adebayo-Tayo and Oscar 2012). The mycotoxin-producing potential of *A. niger, A. versicolor*, and *A. terreus* was confirmed through the fluorescence of these isolates when exposed to UV light on Neutral Red Desiccated Coconut Agar, a method consistent with that of Atanda et al. (2011). The detection of mycotoxins in these fungi emphasises the need for stringent monitoring of infant food products for potential contaminants, as mycotoxins like aflatoxins can cause severe health issues, particularly in infants (Peraica et al. 1999).

The aflatoxin levels detected in the selected infant food samples, ranging from 0.56 ± 0.04 µg/g to 9.20 ± 0.02 µg/g, exceed the regulatory limit of 20 µg/kg set by food safety authorities. This is particularly concerning for infant health, as even low levels of aflatoxins can accumulate over time, leading to chronic health issues (Beretta et al. 2002). For instance, consumption of contaminated cereal by an infant could result in a significant dose of aflatoxins, highlighting the urgency of implementing preventive measures in food production and safety protocols.

Given the risks associated with aflatoxins, the application of *Rhodotorula* yeast pigments as biocontrol agents against fungal pathogens offers a promising alternative to chemical antifungal agents, and since *Rhodotorula* spp. are generally recognised as safe (GRAS) for food applications, with strains such as *R. mucilaginosa* and *R. glutinis* being used in fermented foods and as biocontrol agents (Urbina and Villa-Tanaca 2023). Their safety is attributed to the absence of virulence factors and their long history of use in food systems without adverse effects (Spencer et al. 2021). The three *Rhodotorula* isolates tested in this study produced carotenoid pigments that exhibited significant antifungal activity against *A. versicolor* and *A. niger*. The inhibition zones produced by the yeast pigments (16 mm to 21 mm) were substantially larger than those produced by conventional antifungal drugs, such as fluconazole, ketoconazole, itraconazole, and amphotericin B, which showed minimal activity (1–5 mm). Specifically, YP3 exhibited the highest antifungal activity, with inhibition zones of 21 mm and 19 mm against *A. versicolor* and *A. niger*, respectively.

The observed antifungal properties of *Rhodotorula* pigments are consistent with the findings of Diksha and Kishori (2017), who demonstrated the antimicrobial activity of *Rhodotorula* species against methicillin- and vancomycin-resistant bacteria, as well as with Karanjgaokar and Tarfe (2017), who reported the antifungal effects of *Rhodotorula glutinis* on *Botrytis cinerea* in strawberries. These results reinforce the potential of *Rhodotorula* yeasts and their pigments as natural, environmentally friendly biocontrol agents for reducing fungal contamination in food products.

The use of *Rhodotorula* yeast pigments as an antifungal agent is especially relevant in the context of food safety and sustainability. The use of natural biocontrol agents such as yeast pigments can significantly reduce the reliance on synthetic antifungal chemicals in food preservation, thus minimizing the environmental impact and ensuring safer food products. Moreover, *Rhodotorula* yeasts are generally recognized as safe and have been explored for various applications in the food industry, making them a suitable candidate for incorporation into food safety systems (Sarma et al. 2015).

The carotenoid pigments produced by *Rhodotorula* also provide an added health benefit, as carotenoids are known for their antioxidant properties, which can contribute to overall health and well-being (Márquez et al. 2014). This dual function; antifungal activity and antioxidant properties, makes *Rhodotorula* yeast pigments an attractive alternative to synthetic preservatives, aligning with the goals of sustainable food production and consumption.

Furthermore, the antifungal activity of *Rhodotorula* pigments contributes to the broader goals of reducing mycotoxin contamination in food. The ability to control the growth of toxigenic *Aspergillus* species in cereal-based foods could significantly decrease the risk of aflatoxin contamination, safeguarding the health of infants and other vulnerable populations. This approach aligns with the United Nations Sustainable Development Goal 3 (Good Health and Well-being) and Goal 12 (Responsible Consumption and Production), promoting food safety, sustainability, and the reduction of harmful chemicals in food systems.

## Conclusion

In conclusion, this study demonstrates that *Rhodotorula* yeast pigments exhibit significant antifungal activity against *Aspergillus* species commonly found in cereal-based infant foods. The use of these natural pigments as biocontrol agents presents a promising strategy for reducing fungal contamination and mycotoxin levels in food products, ensuring their safety for infants and other vulnerable consumers. The integration of *Rhodotorula* yeast pigments into food preservation systems, alongside other control measures, could offer a sustainable and effective alternative to synthetic antifungal agents, contributing to more sustainable and environmentally friendly food production practices. Future research should focus on optimising the use of *Rhodotorula* yeast pigments in diverse food matrices and exploring their potential for commercialisation in the food industry.

## Data Availability

Available upon Request.
